# The Association between Physical Activity and Smartphone Addiction in Korean Adolescents: The 16th Korea Youth Risk Behavior Web-Based Survey, 2020

**DOI:** 10.3390/healthcare10040702

**Published:** 2022-04-09

**Authors:** Jooyoung Kim, Kihyuk Lee

**Affiliations:** 1 College of Liberal Arts, Konkuk University, 268, Chungwon-daero, Chungju-si 27478, Korea; jykim1125@kku.ac.kr; 2 Department of Sport Culture, Dongguk University, 30, Phidong-ro 1gil, Jung-gu, Seoul 04620, Korea

**Keywords:** Korea youth risk behavior web-based survey, adolescents, smartphone addiction, overdependence, physical activity, strength exercise

## Abstract

Many side effects of smartphone addiction have been reported, such as a lack of sleep, obesity, and poor concentration. However, the relationship between physical activity (PA) and smartphone addiction has not been fully elucidated. This study aimed to analyze the relationship between physical activity and smartphone addiction among 53,534 Korean adolescents using raw data from the 16th (2020) Korea Youth Risk Behavior Web-based Survey (KYRBS). The dependent variables were the general user group, potential risk user group, and high-risk user group for smartphone addiction. The independent variables were moderate PA (over 5 days per week), vigorous PA (over 3 days per week), and strength exercise (over 3 days per week). Sex, body mass index (BMI), school grade, academic achievement, sleep satisfaction, depression, loneliness, and stress were selected as confounding variables. A complex sample logistic regression analysis was performed. Potential smartphone addiction risk users showed statistically significant odds ratios of 1.423 (*p* < 0.001), 1.379 (*p* < 0.001), and 1.383 (*p* < 0.001) based on general users participating in moderate PA, vigorous PA, and strength exercise, respectively. High-risk users showed statistically significant odds ratios of 1.475 (*p* < 0.001), 1.484 (*p <* 0.001), and 1.619 (*p <* 0.001), respectively. In conclusion, to prevent smartphone addiction among Korean adolescents, participation in moderate PA for more than five days a week, vigorous PA for more than three days a week, or strength exercise for more than three days a week is considered effective.

## 1. Introduction

Smartphones have become an indispensable necessity for modern people because of their various efficiencies and comforts. However, a previous study suggested that the convenience and structural characteristics of a smartphone can consequently cause smartphone addiction [1,2,3]. According to the 2020 Survey on Smartphone Overdependence in Korea, 19.3% of the total population are potential risk users for smartphone overdependence, and 4.0% are high-risk users [4].

When analyzing the risk of smartphone overdependence by generation, 35.8% of adolescents were potential risk (30.8%) and high-risk (5.0%) users of smartphone overdependence [4], indicating that they are more exposed to smartphone addiction than other generations. This is because adolescents lack the ability to control impulsive behavior and are more easily addicted to smartphones than other age groups [5]. Moreover, the number of non-face-to-face classes at each school has increased owing to the recent COVID-19 pandemic, resulting in a decrease in external activities and an increase in smartphone usage time for adolescents in Korea [6]. Accordingly, it was found that the number of adolescents at potential risk of smartphone overdependence in 2020 increased by 5.6% compared to 2019 [7].

Adolescence is an intermediate stage between childhood and adulthood, where social, emotional, and physical growth and development occur rapidly. The establishment of proper lifestyle habits in adolescence is also closely related to lifestyle habits in adulthood. Health conditions in adolescence also affect health in adulthood [8]. Therefore, it is important to have an appropriate lifestyle to promote desirable development and health. Regular PA and exercise not only prevent obesity but also improve academic achievement in adolescents [9]. Despite the advantages of regular PA and exercise, most adolescent PA and exercise have been shown to be insufficient. According to the 2021 Korea Disease Control and Prevention Agency (KDCA), the proportion of adolescents who practice physical activity for 60 min a day more than five days a week was 21.5% for male students and 14.2% for female students [10].

Moreover, adolescents’ sitting time increases annually. It has been reported that the longer the computer usage and TV viewing time at home, the less the physical activity [11]. Consequently, lower physical fitness levels and higher rates of students are suffering from weight gain and sleep disorders [12]. Many previous studies have predicted a decrease in adolescents’ PA due to various factors such as the increase in smartphone use and the development of the internet [13,14]. Smartphone addiction causes physical, emotional, and psychological problems in adolescents because smartphones have fewer time and space restrictions than other media [15].

According to previous studies, young people with higher smartphone overdependence scores showed lower levels of physical fitness [16], were reported to be under a lot of stress [17], and had lower levels of PA [13]. Moreover, it has been reported that adolescent participation in exercise increases self-control, and it decreases loneliness and the risk of smartphone overdependence [18,19]. Another previous study analyzed the influence of PA on smartphone addiction among 110 Chinese international students living in Korea and reported that fewer walking steps per day had a negative correlation with smartphone addiction (*p <* 0.001) [20]. However, previous studies only targeted some middle school students or Chinese international students living in Korea and did not consider variables such as stress, BMI, gender, and school grade. Thus, they cannot represent the relationship between overall Korean adolescents’ smartphone addiction and PA. To clarify the relationship between PA and smartphone addiction, research using samples from many adolescents is required.

The purpose of this study is to analyze the risk of smartphone addiction according to the level of PA of Korean adolescents based on raw data collected through the 16th Korea Youth Risk Behavior Web-based Survey in 2020 and to provide basic data for PA to reduce the risk of smartphone addiction.

## 2. Materials and Methods

### 2.1. Participants and Procedure

This study utilized the KYRBS, which was completed by first-grade middle school students to third-grade high school students in Korea. The KYRBS is an anonymous self-reported online survey that has been conducted annually since 2005 by the KDCA to identify middle and high school students’ health behaviors. This survey is a government-approved statistical survey (Approval No. 11758) that has been conducted annually since 2005 and is based on the National Health Promotion Act (Article 19). The questionnaires consist of 103 items in 16 areas, such as physical activity, mental health, smartphone addiction, and sleep habits. A copy of the questionnaire is attached in the Appendix and can be downloaded online [10]. All data from the survey were obtained from the anonymous self-administered web-based survey. Data from the survey can be used with permission. Additional details about the sampling methodology and survey procedure are available in a previous study [21]. This study was conducted in accordance with the guidelines and regulations provided by the Institutional Review Board of the KDCA. In this study, raw data from the survey conducted in 2020 were used. Personal data were not collected. Sampling and recruitment were targeted at all students in the classes that were selected as sample classes through population stratification, sample distribution, and sampling stages. Those with long-term absence on the day of the survey, special children, and students with textual disabilities were excluded from the survey. The survey period was from 3 August to 13 November 2020. A total of 57,925 students from 800 schools, 400 middle schools, and 400 high schools were targeted, and the final number of participants was 54,948, showing a 94.9% participation rate. In this study, data from 53,534 people were used, excluding responses with missing values for the major variables.

### 2.2. Measures

#### 2.2.1. Independent Variable

The independent variable in this study was related to PA. For PA, a questionnaire on moderate physical activity, vigorous physical activity, and strength exercise was used. The item about moderate PA was, “In the last seven days, how many days did you spend more than 60 min a day on physical activities (regardless of the type) where your heart rate increased, or you were out of breath?” The answers were, “None in the last 7 days”, “1 day a week”, “2 days a week”, “3 days a week”, “4 days a week”, “5 days a week”, and “6 days a week”. Moderate PA was classified as “yes” if it was performed for more than 5 days a week and “no” if it was not. The item about vigorous PA was, “In the last seven days, how many days have you been out of breath or sweating for more than 20 min?” The answers were, “None in the last 7 days”, “1 day a week”, “2 days a week”, “3 days a week”, “4 days a week”, and “More than 5 days a week”. Vigorous physical activity was classified as “yes” if it was performed for more than 3 days a week and “no” if it was not. The item about strength exercise was, “In the last 7 days, how many days have you done muscle strength exercises (muscle strengthening exercises) such as push-ups, sit-ups, lifting weights, dumbbells, pull-ups, and parallel bars?” The answers were, “None in the last 7 days”, “1 day a week”, “2 days a week”, “3 days a week”, “4 days a week”, and “More than 5 days a week”. Strength exercise was classified as “yes” if it was performed for more than 3 days a week and “no” if it was not. In addition, by adding the item of regular PA, a person who completed one or more of the three physical activities previously mentioned(more than 5 days of moderate physical activity, more than 3 days of vigorous physical activity, and more than 3 days of strength exercise) was classified as two answers: “yes” and “no”.

#### 2.2.2. Dependent Variable

The dependent variable in this study was the smartphone addiction scale. Smartphone addiction can be defined in various ways. A previous study described smartphone addiction as a dependent and obsessive behavior caused by excessive smartphone use. Another study defined the term “smartphone overdependence” instead of smartphone addiction as a state in which self-control fails and salience and serious consequences are experienced. Smartphone addiction or overdependence is not a recognized clinical diagnosis. Hence, this study considered addiction and overdependence to have the same meaning in order to reduce confusion regarding the terms. The scale used was developed by the Korea Information Society Agency (2020) [22]. The smartphone addiction questionnaire consisted of self-control failure (three questions), salience (three questions), and serious consequences (four questions). Each item in the questionnaire was rated on a Likert scale. This was calculated with 4 points for “very much”, 3 points for “yes”, 2 points for “not”, and 1 point for “not at all”, with a total score of 40 points. The item scores were summed and classified into general user groups with a total score of 22 or less. A total score of 23–30 was classified as the potential risk user group, and a total score of 31 points or more was classified as the high-risk user group. As a result of the questionnaire reliability analysis, the Cronbach’s alpha coefficients were 914 (total), 937 (control failure), 854 (salience), and 777 (serious consequences).

#### 2.2.3. Confounding Variable

Potential confounding variables that could affect the results of this study were selected by previous studies [16,17,20,22,23,24,25,26,27]. The confounding variables were those that could affect smartphone addiction. Gender, school grade, BMI, academic achievement, sleep satisfaction, depression, loneliness, and stress were analyzed. Among the confounding variables, gender, grade, academic achievement, and depression were used without reconstruction, according to the definition of the 16th KYRBS Index. The answer to sleep satisfaction was classified as “yes”, with “very satisfactory” and “satisfactory” among the answers. The answers of “just so-so”, “unsatisfactory”, and “very unsatisfactory” were classified as “no”. The answers of “I didn’t feel lonely at all”, “I hardly felt lonely”, and “Sometimes I felt lonely” were classified as “I didn’t feel lonely”. The answers of “I often felt lonely” and “I always felt lonely” were classified as “I felt lonely”. The answer to stress was classified as “stress perception”, with “feel very much” and “feel a lot” among the answers. The answers of “I feel a little”, “I don’t feel much”, and “I don’t feel any stress” were classified as “I don’t feel stress at all”.

### 2.3. Statistics

All data were analyzed using SPSS (ver. 25.0). The data of this study presented representative output values for Korean adolescents through a complex sample design. All estimates were calculated based on the sample weights. Consequently, 53,534 middle and high school respondents represented 2,568,334 middle and high school students in the Republic of Korea. The general characteristics of the participants were described, and percentages are presented using a cross-analysis (chi-squared tests) to determine the relationship between PA and smartphone dependence. The relationship between PA and smartphone dependence was analyzed using a polynomial logistic regression analysis, where correction variables were used to exclude the effects of confounding variables, and odds ratios (OR) and 95% confidence intervals (CI) were calculated.

## 3. Results

### 3.1. General Characteristics of the Survey Subjects and the Results of Cross-Analysis

The general characteristics of the participants in this study were as follows: 53,534 students from the first grade of middle school to the third grade of high school comprised the participants of the survey. They consisted of 40,130 (75.0%) general smartphone users, 11,747 (22.1%) users at potential risk for smartphone addiction, and 1,557 (2.9%) users at high-risk for smartphone addiction. As a result of the cross-analysis, to determine the association between general characteristic variables and smartphone dependence, there was a significant difference between smartphone addiction among male and female students (*p* < 0.001). The confounding variables of BMI, school grade, academic achievement, depression, loneliness, and stress all differed significantly from smartphone addiction (*p* < 0.001–< 0.01). In the case of PA, which is an independent variable, moderate PA, vigorous PA, strength exercise, and regular PA all resulted in significant differences in smartphone addiction (*p* < 0.001). Table 1 and Table 2 show the results of the detailed cross-analysis of smartphone addiction and each variable.

### 3.2. Relationship between Moderate Physical Activity and Smartphone Addiction

Table 3 shows the relationship between moderate PA and smartphone addiction using the odds ratio. Model 1 represents an uncorrected output value, and Model 2 represents the value analyzed by correcting the potential confounding variables. Based on the general users who participated in moderate PA for more than five days a week in Model 1, the possibility of smartphone addiction among potential risk users who did not participate in moderate PA at all was 1.560 times higher (*p* < 0.001) and that of high-risk users was 1.686 times higher (*p* < 0.001). In Model 2, based on students participating in moderate PA for more than five days a week, the possibility of smartphone addiction among potential risk users who did not participate in moderate PA was 1.423 times higher (*p* < 0.001) and that of high-risk users was 1.475 times higher (*p <* 0.001).

### 3.3. Relationship between Vigorous Physical Activity and Smartphone Addiction

Table 4 shows the relationship between vigorous PA and smartphone addiction using the odds ratio. Based on general users who perform vigorous PA for more than three days a week in Model 1, potential risk users who do not perform vigorous PA at all were 1.513 times (*p* < 0.001) more likely to be addicted to smartphones and high-risk users 1.702 times higher (*p* < 0.001). In Model 2, the possibility of smartphone addiction was 1.379 times (*p <* 0.001) and 1.484 times (*p* < 0.001) higher, respectively.

### 3.4. Relationship between Strength Exercise and Smartphone Addiction

Table 5 shows the relationship between strength exercise and smartphone addiction using the odds ratio. In the case of strength exercise, Model 1 shows that potential risk users who do not perform weight training at all were 1.536 times (*p* < 0.001) more likely to be addicted to smartphones. Based on general users who perform weight training for more than three days a week, high-risk users were 1.924 times (*p* < 0.001) more likely to be addicted to smartphones. In Model 2, the possibility of smartphone addiction was 1.383 times (*p* < 0.001) and 1.619 times (*p* < 0.001) higher, respectively.

### 3.5. Relationship between Regular Physical Activity and Smartphone Addiction

Table 6 shows the relationship between regular PA and smartphone addiction using the odds ratio. Regular PA was defined as participation in regular PA if one or more of the three PAs (moderate PA, vigorous PA, and strength exercises) were completed. The relationship between regular PA participation and smartphone addiction (odds ratio) is presented in Table 5. In the case of regular PA, Model 1 indicated a 1.512 times higher risk of smartphone addiction in potential risk users who did not perform regular PA at all (*p* < 0.001) and a 1.851 times higher risk for high-risk users (*p* < 0.001). In Model 2, the possibility of smartphone addiction was 1.351 times (*p* < 0.001) and 1.549 times (*p* < 0.001) higher, respectively.

## 4. Discussion

Many side effects (a lack of sleep, obesity, and poor concentration) of smartphone addiction have been reported [27,28,29]. Previous studies have shown that the higher the smartphone addiction score, the lower the physical fitness [16]. However, the relationship between PA and smartphone addiction has not been fully elucidated. The purpose of this study was to analyze the risk of smartphone addiction according to the level of PA of Korean adolescents based on raw data collected through the 16th KYRBS in 2020. Our study included the largest sample of Korean adolescents to date to investigate the prevalence of smartphone addiction and clarify the association between PA and smartphone addiction.

As a result of cross-analysis of confounding variables that are considered to affect the level of smartphone addiction, all confounding variables differed significantly from the level of smartphone addiction. Between smartphone addiction and gender, female adolescents comprised a higher percentage of high-risk smartphone users than male adolescents (63.2% vs. 36.8%). This is similar to previous studies that compared the difference in the level of smartphone addiction between males and females [30]. Additionally, this seems to reflect the characteristics of female adolescents, who are more interested in the media and prefer static activities [31]. As a result of the cross-analysis of smartphone addiction, in which BMI was divided based on the index of 23, users with a BMI of 23 or higher comprised the highest percentage of high-risk users (*p* < 0.05). As a result of analyzing BMI and the level of smartphone addiction in previous studies, it was found that the higher the smartphone addiction score, the higher the probability of a high BMI [16], which is consistent with our research results. As for the level of smartphone addiction according to grade, the 7th grade comprised the highest percentage of general smartphone users, and the 11th grade comprised the highest percentage of potential risk users and high-risk users. This suggests that there is an increase in the risk of smartphone addiction the higher the grade. However, it seems that students refrain from using smartphones in the 12th grade (high school senior) ahead of college entrance. In the case of the academic achievement of high-risk users, adolescents who responded that their academic achievement was low were the most likely to be addicted to smartphone. This result is similar to those of previous studies in which it was reported that the risk of smartphone addiction had a negative effect on academic achievement [26]. Adolescents who responded that they experienced depression, loneliness, and stress comprised the highest percentage in the high-risk group for smartphone addiction.

In this study, a cross-analysis was conducted to assess the difference between the intensity of PA and strength exercise according to the risk of smartphone addiction. The results show that moderate PA, vigorous PA, strength exercise, and regular PA all significantly differ according to the risk of smartphone addiction. Moreover, the risk of smartphone addiction increased by 1.429 times in the potential risk group and 1.478 times in the high-risk group based on adolescents who performed moderate PA for more than 5 days a week. In case of vigorous PA 3 days per week, the risk of smartphone addiction increased by 1.385 times in the potential risk group and by 1.488 times in the high-risk group. In the case of strength exercises, the risk of smartphone addiction increased by 1.387 times in the potential risk group and by 1.621 times in the high-risk group. In case of regular PA, the risk of smartphone addiction increased by 1.357 times in the potential risk group and that of the high-risk group increased by 1.553 times. A study on the relationship between smartphone addiction and PA among Chinese international students living in Korea showed that a high-risk group of smartphone addiction students had low daily walking steps (*p* < 0.001) compared with a no-risk group [20]. In addition, it was reported that the amount of fat mass was larger (*p* < 0.001) and the amount of muscle mass was smaller in the high-risk group (*p* < 0.001). Another previous study analyzed the relationship between smartphone addiction and physical strength in middle school students and found that the higher the smartphone addiction score, the lower the physical fitness (*p* < 0.05) [16]. This is consistent with our results, which show that the presence or absence of various PAs, including strength exercises, increases the risk of smartphone addiction. Moreover, the excessive use of smartphones interferes with PA [13], and considering that low energy consumption due to inactivity is related to various health problems, including obesity or metabolic syndrome [32,33], PA and exercise intervention are necessary to prevent smartphone addiction. Long-term and continuous PA has been reported to increase the risk of high blood pressure in the case of young subjects [34]. Furthermore, another previous study reported that students with extremely high PA as well as low PA showed significantly higher sleepiness than those with moderate PA [35]. Therefore, in order to care for one’s health, attention should be paid to an excessive increase in PA as well as a decrease in PA.

This study collected data from a large sample of Korean adolescents using verified and widely used smartphone addiction scales. We conducted a survey of 53,534 Korean adolescents, collected data by applying complex sampling, and applied weights to represent the entire youth population in Korea in 2020. Our study dealt with a new and remarkable phenomenon in the relationship between PA and smartphone addiction, and it was found that a lack of PA increases the risk of smartphone addiction. However, this study has several limitations. Owing to the cross-sectional nature of the data collection, the possibility of an inverse causal relationship cannot be ignored. It is important to pay attention to the estimation of PA because it only relies on the responses of the participants in the evaluation of PA. Therefore, further studies require more specific measurements of PA to verify the relationship between smartphone addiction and PA. In addition, research on improving smartphone addiction through PA interventions is required. 

## 5. Conclusions

The level of smartphone addiction among Korean adolescents varies depending on gender, BMI, grade, academic achievement, depression, loneliness, and stress. Moreover, the absence of regular PA increases the probability of smartphone addiction. Moderate PA for at least 5 days a week, vigorous PA for at least 3 days a week, or muscle exercise for at least 3 days a week is recommended to prevent Korean adolescents from becoming addicted to smartphones.

## Figures and Tables

**Table 1:** General characteristics of the participants based on level of smartphone addiction.

	Total User (N = 53,534)			*χ*^2^ (*p*-Value)
	General User (N = 40,130; 74.6%)	Potential Risk User (N = 11,847; 22.5%)	High-Risk User (N = 1557; 2.9%)	
Sex				
Male	21,990 (54.9)	5131 (44.2)	566 (36.8)	570.380
Female	18,140 (45.1)	6716 (55.8)	991 (63.2)	<0.001
BMI				
<23	27,558 (69.2)	8464 (71.5)	1126 (72.6)	28.138
23≤	12,572 (30.8)	3383 (28.5)	431 (27.4)	<0.001
School grade				
7th	7934 (19.6)	1684 (13.3)	182 (11.7)	360.644
8th	7033 (16.4)	2035 (15.7)	263 (15.3)	<0.001
9th	6642 (15.0)	2197 (16.9)	322 (19.4)	
10th	6499 (16.8)	1975 (17.4)	213 (14.0)	
11th	6255 (16.4)	2109 (18.9)	305 (20.4)	
12th	5767 (15.8)	1847 (17.7)	272 (19.2)	
Academic achievement				
High	5337 (13.3)	1095 (9.1)	140 (8.9)	734.485
Middle high	10,246 (25.8)	2662 (22.5)	287 (18.7)	<0.001
Middle	12,417 (30.8)	3460 (29.3)	364 (23.5)	
Middle low	8672 (21.4)	3197 (27.1)	418 (27.1)	
Low	3458 (8.6)	1433 (11.9)	348 (21.8)	
Sleep satisfaction				
Yes	13,418 (33.1)	3995 (22.4)	768 (20.2)	578.007
No	31,497 (66.9)	7852 (77.6)	789 (79.8)	<0.001
Depression				
Yes	8633 (21.5)	3995 (33.5)	768 (49.4)	1224.416
No	31,497 (78.5)	7852 (66.5)	789 (50.6)	<0.001
Loneliness				
Yes	5608 (14.0)	1672 (14.1)	220 (14.4)	0.233
No	34,522 (86.0)	10,175 (85.9)	1337 (85.6)	0.004
Stress				
Yes	12,030 (30.2)	5086 (42.7)	929 (59.9)	1132.303
No	28,100 (69.8)	6761 (57.3)	628 (40.1)	<0.001

BMI: body mass index. Values are N (%). Percentage is weighted.

**Table 2:** Difference in physical activity based on level of smartphone addiction.

	Total User (N = 53,534)			*χ*^2^ (*p*-Value)
	General User (N = 40,130)	Potential Risk User (N = 11,847)	High-Rik User (N = 1557)	
Moderated PA				
Yes	9062 (21.6)	1812 (15.0)	229 (14.0)	286.256
No	31,068 (78.4)	10,035 (85.0)	1328 (86.0)	<0.001
Vigorous PA				
Yes	7951 (18.8)	1610 (13.3)	193 (12.0)	229.563
No	32,179 (81.2)	10,237 (86.7)	1364(88.0)	<0.001
Strength exercise				
Yes	15,021 (36.6)	3281 (27.3)	371 (23.1)	442.303
No	25,109 (63.4)	8566 (72.7)	1186(76.9)	<0.001
Regular PA				
Yes	17,894 (43.6)	4059 (33.8)	467 (29.4)	454.254
No	22,236 (56.4)	7788 (66.2)	1090 (70.6)	<0.001

PA: physical activity. Values are N (%). Percentage is weighted.

**Table 3:** Logistic regression analysis on level of smartphone addiction based on moderate PA.

		Model 1		Model 2	
Moderated PA		OR (95% CI)	*p*-Value	OR (95% CI)	*p*-Value
Potential risk user	Yes	Reference	-	Reference	-
	No	1.560 (1.547–1.572)	<0.001	1.423 (1.411–1.435)	<0.001
High-risk user	Yes	Reference	-	Reference	-
	No	1.686 (1.651–1.721)	<0.001	1.475 (1.444–1.507)	<0.001

PA: physical activity; Model 1 non-adjusted; Model 2 adjusted for sex, school grade, academic achievement, sleep satisfaction, depression, loneliness, and perceived stress.

**Table 4:** Logistic regression analysis on level of smartphone addiction based on vigorous PA.

		Model 1		Model 2	
Vigorous PA		OR (95% CI)	*p*-Value	OR (95% CI)	*p*-Value
Potential risk user	Yes	Reference	-	Reference	-
	No	1.513 (1.500–1.526)	<0.001	1.379 (1.367–1.391)	<0.001
High-risk user	Yes	Reference	-	Reference	-
	No	1.702 (1.664–1.740)	<0.001	1.484 (1.450–1.518)	<0.001

PA: physical activity; Model 1 non-adjusted; Model 2 adjusted for sex, school grade, academic achievement, sleep satisfaction, depression, loneliness, and perceived stress.

**Table 5:** Logistic regression analysis on level of smartphone addiction based on strength exercise.

		Model 1		Model 2	
Strength Exercise		OR (95% CI)	*p*-Value	OR (95% CI)	*p*-Value
Potential risk user	Yes	Reference	-	Reference	-
	No	1.536 (1.526–1.546)	<0.001	1.383 (1.374–1.393)	<0.001
High-risk user	Yes	Reference	-	Reference	-
	No	1.924 (1.892–1.958)	<0.001	1.619 (1.589–1.649)	<0.001

PA: physical activity; Model 1 non-adjusted; Model 2 adjusted for sex, school grade, academic achievement, sleep satisfaction, depression, loneliness, and perceived stress.

**Table 6:** Logistic regression analysis on level of smartphone addiction based on regular PA.

		Model 1		Model 2	
Regular PA		OR (95% CI)	*p*-Value	OR (95% CI)	*p*-Value
Potential risk user	Yes	Reference	-	Reference	-
	No	1.512 (1.503–1.522)	<0.001	1.351 (1.342–1.360)	<0.001
High-risk user	Yes	Reference	-	Reference	-
	No	1.851 (1.822–1.881)	<0.001	1.549 (1.523–1.576)	<0.001

PA: physical activity; Model 1 non-adjusted; Model 2 adjusted for sex, school grade, academic achievement, sleep satisfaction, depression, loneliness, and perceived stress.

## Data Availability

Releasing of the data by the researchers is not legally permitted. All data are available from the database of the KDCA.
